# Dynamic changes of brain networks during standing balance control under visual conflict

**DOI:** 10.3389/fnins.2022.1003996

**Published:** 2022-10-05

**Authors:** Guozheng Wang, Yi Yang, Jian Wang, Zengming Hao, Xin Luo, Jun Liu

**Affiliations:** ^1^College of Biomedical Engineering and Instrument Science, Zhejiang University, Hangzhou, China; ^2^Department of Sports Science, College of Education, Zhejiang University, Hangzhou, China; ^3^Department of Rehabilitation Medicine, The First Affiliated Hospital, Sun Yat-sen University, Guangzhou, China

**Keywords:** electroencephalography, sensory conflict, standing balance control, effective connectivity, virtual reality

## Abstract

Stance balance control requires a very accurate tuning and combination of visual, vestibular, and proprioceptive inputs, and conflict among these sensory systems may induce posture instability and even falls. Although there are many human mechanics and psychophysical studies for this phenomenon, the effects of sensory conflict on brain networks and its underlying neural mechanisms are still unclear. Here, we combined a rotating platform and a virtual reality (VR) headset to control the participants’ physical and visual motion states, presenting them with incongruous (sensory conflict) or congruous (normal control) physical-visual stimuli. Further, to investigate the effects of sensory conflict on stance stability and brain networks, we recorded and calculated the effective connectivity of source-level electroencephalogram (EEG) and the average velocity of the plantar center of pressure (COP) in healthy subjects (18 subjects: 10 males, 8 females). First, our results showed that sensory conflict did have a detrimental effect on stance posture control [sensor *F*(1, 17) = 13.34, *P* = 0.0019], but this effect decreases over time [window*sensor *F*(2, 34) = 6.72, *P* = 0.0035]. Humans show a marked adaptation to sensory conflict. In addition, we found that human adaptation to the sensory conflict was associated with changes in the cortical network. At the stimulus onset, congruent and incongruent stimuli had similar effects on brain networks. In both cases, there was a significant increase in information interaction centered on the frontal cortices (p < 0.05). Then, after a time window, synchronized with the restoration of stance stability under conflict, the connectivity of large brain regions, including posterior parietal, visual, somatosensory, and motor cortices, was generally lower in sensory conflict than in controls (p < 0.05). But the influence of the superior temporal lobe on other cortices was significantly increased. Overall, we speculate that a posterior parietal-centered cortical network may play a key role in integrating congruous sensory information. Furthermore, the dissociation of this network may reflect a flexible multisensory interaction strategy that is critical for human posture balance control in complex and changing environments. In addition, the superior temporal lobe may play a key role in processing conflicting sensory information.

## Introduction

Stance balance control is a complex process in which the brain must continuously combine multisensory information from the visual, vestibular, and somatosensory systems to activate muscles for proper posture or movement. Vision plays an important role in the control of posture, and when these three sensory systems are isolated and tested for balance, it has been found that vision is the most important contributor to balance (Hansson et al., 2010). However, the source of visual motion is inherently ambiguous, and the movement of objects in the environment and self-movement can cause similar visual stimuli (Fushiki et al., 2005). In some complex environments, the conflict between visual and nonvisual information may lead to unstable posture, vertigo, and even falls (Keshavarz et al., 2015). Theoretically, the brain could mitigate this problem by combining visual signals with other types of information. Human mechanics studies have shown that the integration of sensory information used for postural control appears dynamically regulated to adapt to changing environmental conditions and available sensory information (Peterka and Loughlin, 2004; Mahboobin et al., 2009). And numerous psychophysical studies have shown that during multisensory processing, the brain needs to make judgments about the source of multimodal sensory cues, arbitrating between sensory integration, and segregation (Dokka et al., 2010, 2019; Shams and Beierholm, 2010; Acerbi et al., 2018; Rohe et al., 2019). It performs weighted fusion for sensory signals from common sources and separate processing for sensory information from independent sources (Ernst and Banks, 2002; Fetsch et al., 2009).

The exact neural mechanisms of multisensory interactions are still unclear, but several cortical regions have been identified as a crucial contribution (Bolton, 2015). Neurophysiological studies in macaques have shown that multisensory information is combined in the dorsal division of the medial superior temporal area and the ventral intraparietal area. Both areas contain neurons that show selectivity for optic flow patterns and directional tuning for inertial motion in darkness (Tanaka et al., 1986; Duffy and Wurtz, 1991; Page and Duffy, 2003; Gu et al., 2006), and some of these neuronal populations are sensitive to congruent stimuli and others to incongruent stimuli. Neuroimaging studies in humans have also localized multiple multisensory cortices, including numerous areas in the parietal cortex (e.g., the ventral intraparietal area), temporal cortex (e.g., the caudal superior temporal polysensory region), and frontal cortex. Further, congruency of sensory cues was also found to affect cortical activation, with stronger activation in the posterior insula and temporal lobes during sensory conflict and increased activation in the primary and secondary visual cortex when sensory cues were congruent (Roberts et al., 2017); Hemodynamic studies have shown that medial temporal and medial superior temporal regions have stronger activation in conflict conditions compared to congruent conditions (Nguyen et al., 2020). In addition, the subjective strength of the vertiginous sensation was negatively correlated with the hemodynamic activity of the intraparietal sulcus and supramarginal gyrus.

Although previous studies have identified many cortical regions that are significantly activated during multisensory interactions, the interactions within and between the relevant cortical regions remain to be investigated. It is well known that multisensory processing requires the coordinated activity of different cortical areas, and key mechanisms involved in these processes include local neural oscillations and connections between distant cortical areas (von Stein and Sarnthein, 2000; Keil and Senkowski, 2018). Therefore, it is important to study the flow of information across multiple cortical areas that process and integrate sensory cues. Traditional neuroimaging methods are limited by fixed recordings and low temporal resolution, such as functional magnetic resonance imaging (fMRI) and functional near-infrared spectroscopy (fNIRS). In contrast, electroencephalography (EEG) is a promising method for assessing human cortical interactions during dynamic homeostasis due to its high temporal resolution and portability (Gramann et al., 2011). Although EEG may be limited by low spatial resolution and artifact contamination, cortical activity can be obtained by source analysis techniques while removing artifacts from it by blind source separation techniques such as independent component analysis (ICA), thereby improving spatial resolution and reducing artifact contamination (Gwin et al., 2010).

In the present study, we hypothesized that cross-modal differences in sensory information affect the brain’s strategies for multisensory processing in stance balance control and that this can be revealed by quantitative measures of interactions between relevant cortical regions. Here, according to our working hypothesis, we combined a rotating platform and a virtual reality (VR) headset to control participants’ body movements and visual information to construct incongruous (sensory conflict) or congruous (normal control) physical-visual rotational stimuli. And the information flow in the cortical network was measured quantitatively by partial directed coherence (PDC, a time-varying, frequency-selective, and directional functional connectivity analysis tool) (Baccala and Sameshima, 2001; Friston, 2011).

## Materials and methods

### Participants

Twenty-two college students participated in this study. Subjects were volunteers from the local university campus who responded to a network advertisement. All participants had no neurological, skeletal, or muscular problems and normal or corrected vision and signed a written informed consent form. The Ethics Committee of Zhejiang University Psychological Science Research Center permitted our experiment (issued no. 2020-003). Four participants were excluded from data analysis because of strides/stumbles during the experiment. Finally, data from 18 participants [10 males, 8 females, age 24.1 ± 2.3 years (mean ± *SD*)] were involved in the statistical analysis.

### Experimental design

In this study, we used a rotating platform and a VR headset to control the participants’ body movements and visual information. The rotation platform (All Controller, Nanjing, P. R. China) can rotate counterclockwise and clockwise with a maximum rotation speed of 96 deg/s. The VR headset used the VIVE Pro Eye wireless kit produced by HTC, which can present a simulated laboratory environment through the Unity3D program (Outside of the programmed scene movement, normal head movements are also picked up by the IMU in the VR headset and change the perspective of the virtual scene accordingly). We used the written Unity3D program to control both the rotating platform and the visual scene in the VR headset, setting both “congruent” (visual information synchronized with the actual body movement) and “incongruent” (visual information conflicting with the actual body movement) experimental conditions.

In the congruent condition, we did not provide additional control in the VR headset. In this case, the participant moved clockwise with the rotating platform at 30°/s relative to the ground, while that motion was picked up by the IMU in the VR headset, and the visual scene in his eyes moved counterclockwise relative to him at 30°/s (just as in a natural rotation in a stationary environment, the visual scene should be stationary relative to the ground, but moving in the opposite direction relative to ourselves). In the incongruent condition, our goal is to construct a scene that should be seen with counterclockwise motion at the same speed in the case of clockwise motion. This that the visual scene provides a message in the opposite direction of the actual motion, with the same intensity. In this case, we added an additional 60°/s of clockwise motion to the scene of the VR headset. At this point, the participant’s body moves clockwise at 30°/s, and the visual scene in his eyes also moves clockwise at 30°/s. In both the congruent and incongruent conditions, the platform and the visual scene began to move after 30 s of standing and stopped after 36 s of uniform rotation at 30 deg/s; The acceleration and deceleration processes were completed within 1 s; Participants were then asked to continue standing quietly for 34 s, and then rest for 5 min waiting for the next test task.

**FIGURE 1:**
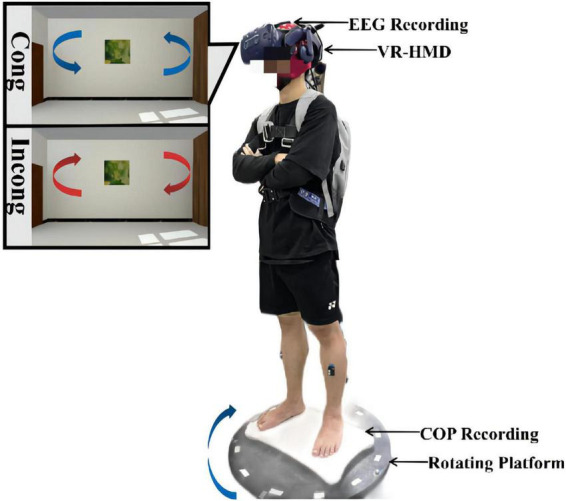
Schematic diagram of the experimental setup and installation. The experimental system was composed of an EEG acquisition device, a VR headset, a force measurement platform, and a rotating platform. During the experiment, the participants stood on the force measurement platform and were asked to look straight ahead with their hands on their chests the whole time. Both physical and visual distractions were applied through the rotating platform and the VR headset. The rotation was always clockwise, while the visual scene was rotated counterclockwise at the same speed in the congruent condition and clockwise at the same speed in the incongruent condition.

The experimental setup and settings are shown in Figure 1. Before the start of the formal test, we informed the participants of the experimental procedure, and all participants familiarized themselves with each condition. In the formal test, each participant was asked to stand on the force plate with arms crossed over the chest in a shoulder-width position. All subjects were instructed to keep their eyes open (normal blinks were allowed) and look straight ahead throughout the experiment, which was ensured by an eye-tracking system built into the VR headset. The force plate was placed in the center of the rotating platform. Next, all participants were asked to maintain standing balance to complete three test tasks (stationary standing baseline, congruent and incongruent physical-visual stimuli). The order in the three tasks was randomized and had 5 min of rest between different test tasks. In the standing baseline condition, the rotating platform and the VR scene remained motionless for 100 s.

### Data collection and preprocessing

In this study, we mainly collected plantar center of pressure (COP) data and EEG data from participants during the experimental task. We placed the Wii balance board in the center of the rotating platform and measured the subjects’ COP under the three experimental tasks with a sampling rate of 100 Hz. Many studies have verified the validity and reliability of the Wii balance board in COP measures (Clark et al., 2010; Huurnink et al., 2013; Leach et al., 2014). The validity of the COP data in this study is discussed in Supplementary Figure 1. The EEG signals were acquired with the ANTNeuro EEG device, containing 32 channels in the 10–20 standard regime, and the impedance of all electrodes was kept below 5 k ohms throughout the experiment, at a sampling rate of 1,000 Hz sampling.

We preprocessed the COP and EEG data in a custom script in Matlab. First, we discard the signals in the acceleration or deceleration phase of the rotation platform and then divide the COP data and EEG data into six phases: baseline (BS, 12 s before platform start), rotating1 (R1, 0–12 s after platform start), rotating2 (R2, 12–24 s after platform start), rotating3 (R3, 24–36 s after platform start), after1 (A1, 0–12 s after platform stop), and after2 (A2, 12–24 s after platform stop). The COP data were filtered using a 20 Hz low-pass, 2nd order, zero-lag Butterworth filter. Furthermore, the mean of the filtered data was removed.

Sway area quantifies 85% of the total area covered in the ML and AP directions using an ellipse to fit the COP data, which is considered an index of overall postural performance (Palmieri et al., 2002; Paillard and Noe, 2015). Sway mean velocity is calculated by dividing the COP trajectory distance by the duration, which is considered to be the reliable dynamic indicator of the efficiency of posture control (the smaller the speed, the better the posture control) (Paillard and Noe, 2015; Luo et al., 2018). We calculated mean sway velocity of the COP signal as follows:


$$M\,V=\frac{\sum_{\text{i}=1\;}^{N}{\sqrt{{\left(x_{\text{i}+1\;}-\;x_{i}\right)}^{2}\;+\;{\left(y_{i+1}\;-\;y_{i}\right)}^{2}}}^{*}F}{N}$$

where x(i) and y(i) are the COP displacements in the ML and AP directions, respectively, N = number of samples, F = sampling frequency.

**FIGURE 2:**
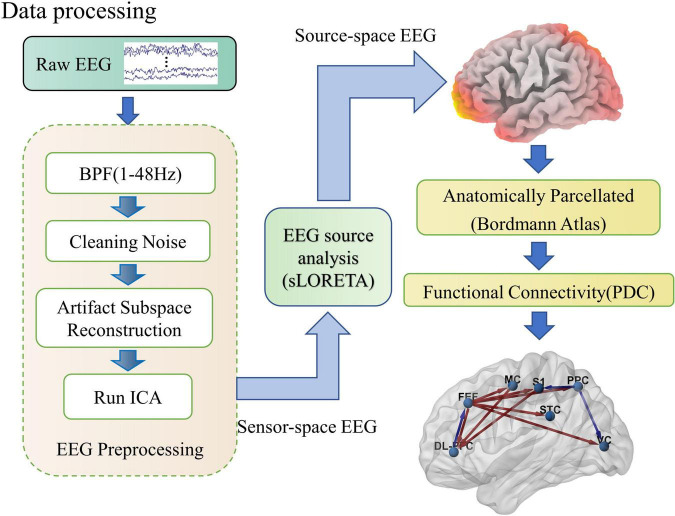
Schematic diagram of the EEG processing flow. As shown in the figure, EEG processing includes three parts: preprocessing, soliton source analysis, and network connection calculation. In the preprocessing, raw EEG is cleaned up by band-pass filtering, removal of linear interference, artifact spatial reconstruction, and independent component analysis steps. Then the sensor space EEG is transformed into source activity and clustered into regions of interest by the sLORETA software package. Finally, the effective connections between cortical regions of interest are calculated quantitatively using PDC.

We processed the EEG data mainly based on a custom script in the EEGLAB toolbox (Delorme and Makeig, 2004), it was first band-pass filtered from 1 to 48 Hz, and the 50 Hz line noise was removed using the EEGLAB Cleanline plug-in. Further, to remove artifacts from body motion during rotation, the EEG data segments contaminated with large artifacts were removed using Artifact Subspace Reconstruction (ASR) (Mullen et al., 2015), where the threshold was set to 20 standard deviations and ensured that at least 62.5 percent (Every 12 s data is guaranteed to leave 7.5 s) of the data were retained. Finally, the EEG signal was decomposed using ICA with the aid of the ICLabel plug-in to remove interfering signals such as blinks, muscle artifacts, electrocardiogram, and linear noise that are not homologous to the EEG (Pion-Tonachini et al., 2019). The processing flow of EEG data is shown in Figure 2.

### Electroencephalogram sourcing analysis and connectivity computation

The EEG source analysis allows us to study cortical dynamics from the potential cortical source activity estimated from the sensor space EEG. Here, we used a standardized low-resolution EEG tomography software package for source localization (Pascual-Marqui, 2002). First, a head model (forward model) was created using a Boundary Element Method based on the ICBM152 brain anatomy (Fuchs et al., 1998; Mazziotta et al., 2001). Inverse modeling based on a standardized low-resolution electromagnetic tomography algorithm was used to compute cortical time series data. The sources were restricted to the cortex, and the reconstructed time series were projected to the region of interest (ROI) defined by the Brodmann atlas (Zilles and Amunts, 2010). Specifically, we selected the following seven cortical regions as ROIs: dorsolateral prefrontal cortex (DL-PFC; BA10, 46, 47), frontal eye field cortex (FEF; BA8, 9), motor cortex (MC; BA4, 6), primary somatosensory (S1; BA1, 2, 3), posterior parietal cortex (PPC; BA5, 7), superior temporal cortex (STC; BA22, 40), visual cortex (VC; BA17, 18, 19).

After obtaining the activity of the seven cortical regions of interest, we used PDC to perform an effective connectivity analysis (Baccala and Sameshima, 2001). PDC, a frequency domain extension of Granger causality, can be used to calculate the strength of directed information flow between cortical regions. We used the open-source toolbox HERMES to calculate PDC for theta (4–8 Hz) and alpha (8–12 Hz) bands, where the time window was set to 3 s with 50% overlap (Each segment of 7.5 s of valid data is divided into 4 windows), and the model order p was determined using the Akaike information criterion. Then node strength was computed as the sum of weights of links connected to the node.

### Statistical analysis

First, to determine the effect of sensory conflict on stance stability, we used two-way repeated measures ANOVA to assess the effects of the time window and sensory condition on COP sway velocity. Differences in COP sway velocity within rotating phase (R1, R2, R3) or after rotating phase (A1, A2) were assessed with two-way repeated measures ANOVAs with sensory condition (incongruent, congruent) and time window (per 12 s) factor. Differences in COP sway velocity during the movement state transitions were assessed with repeated measures ANOVAs, which compared the baseline to the mean of the rotating and after rotating phases in both sensory conditions. In ANOVAs, predicted effects and/or interactions were explored further with simple effects analyses, and unexpected effects were explored further with Bonferroni *post-hoc* tests.

Further, in order to know the effect of incongruent and congruent rotational stimuli on brain networks, we used paired *t*-tests to analyze the differences in network connection and node strength between two rotational stimuli to baseline. Further, we examined the effect of time windows (R1, R2, and R3) and sensory status on node strength using two-way repeated measures ANOVA and Bonferroni *post-hoc* tests for data from the interference period. In the analysis of cortical connections, p < 0.05 was defined as a significant difference, and all *p*-values were corrected for the false discovery rate (FDR) method (Benjamini and Hochberg, 1995). Significant connections corresponding to p < 0.05 were plotted onto MRI templates using the BrainNet Viewer toolbox (Xia et al., 2013), where red represents significantly stronger, and blue represents significantly weaker. Studentized residuals were tested for normality by Shapiro-Wilk’s test, and non-normal data were analyzed using the Wilcoxon signed-rank test or Friedman two-way analysis of variance.

## Results

### Effect of sensory conflict on standing balance

**FIGURE 3:**
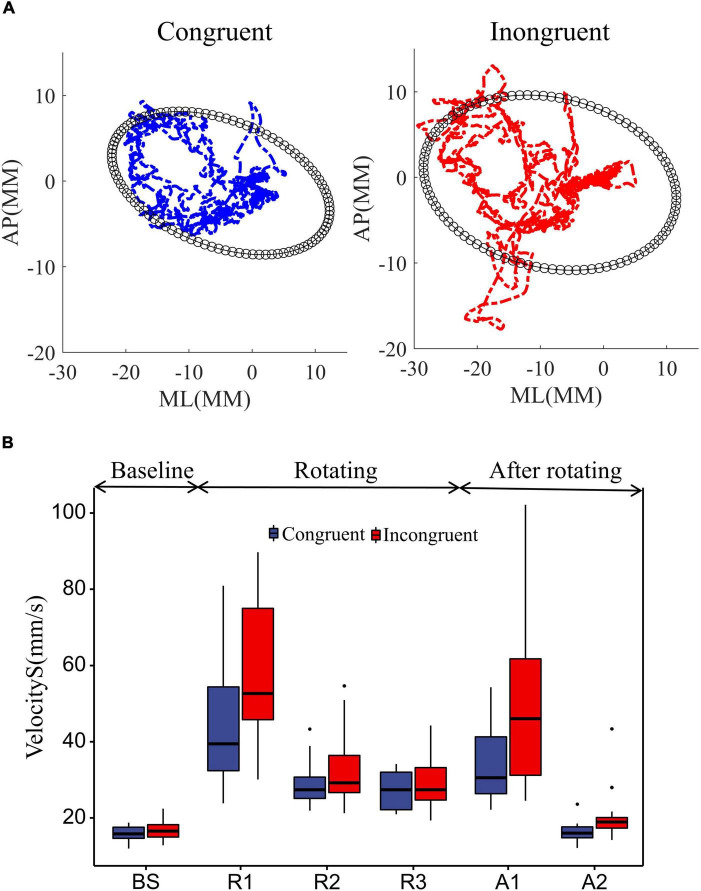
Effect of rotational stimuli on stance stability. **(A)** The mean COP sway trajectories and area for all 18 participants. The blue and red curves represent the mean COP trajectories in the congruent and incongruent conditions, respectively. The black ellipse quantifies 85% of the total area covered in the ML and AP directions, using the ellipse to fit the data. **(B)** Comparative analysis of participant sway velocities between congruent and incongruent conditions. According to the starting and ending points of the rotating stimuli, we divided the COP data into BS (12 s before platform initiation), R1 (0–12 s after platform initiation), R2 (12–24 s after platform initiation), R3 (24–36 s after platform initiation), and A1 (0–12 s after platform stop), A2 (12–24 s after platform stop). We used two-way repeated measures ANOVA to assess the effects of the time window and sensory condition on standing posture stability. Differences in COP sway velocity within rotating phase (R1, R2, R3) and after rotating phase (A1, A2) were assessed with two-way repeated measures ANOVAs with sensory condition (incongruent, congruent) and time window. Differences in COP sway velocity during the movement state transitions were assessed with repeated measures ANOVAs, which compared the baseline to the mean of the rotating and after rotating phases in both sensory conditions. In ANOVAs, predicted effects and/or interactions were explored further with simple effects analyses, and unexpected effects were explored further with Bonferroni *post-hoc* tests. The upper and lower error bars of the bins are the upper and lower quartiles of the data, respectively.

To investigate the effect of sensory conflict on standing postural stability, we collected participants’ center of plantar pressure (COP) in both sensory conditions and recorded their trajectories (Figure 3A). Using a paired *t*-test showed that the range of COP sway was significantly greater in incongruent condition than in congruent condition (*P* = 0.0019). This suggests that sensory conflict has a detrimental effect on stance stability.

Further, to investigate the dynamic effects of sensory conflict on standing posture stability, we used two-way repeated measures ANOVA to assess the effects of the time window and sensory condition on standing posture stability, as shown in Figure 3B. First, the sway velocity increased substantially in both sensory conditions after the platform started to rotate (Figure 3B). A repeated measures ANOVA contrasting average sway velocity at the two windows of baseline with an average of three windows in rotation period confirmed it [window *F*(1, 17) = 108.14, *P* = 8.7e-11], and also sway velocity significantly greater in the incongruent condition than in the congruent condition [sensor *F*(1, 17) = 12.11, *P* = 0.0029].

Then, we analyzed the sway velocity during the rotation duration (R1, R2, R3) using repeated measures ANOVA. The results showed that the sway speed of participants decreased significantly over time [window *F*(2, 34) = 49.38, *P* = 8.8e-11], which reflected the adaptation of the humans to the balance disturbance. And the sway velocity was significantly greater in the incongruent condition than in the congruent condition [sensor *F*(1, 17) = 13.34, *P* = 0.0019], which reflected the detrimental effect of sensory conflict on the standing balance. More importantly, we found a significant interaction effect of the two factors [window*sensor *F*(2, 34) = 6.72, *P* = 0.0035]. This implies that the detrimental effect of sensory conflict on stance stability diminishes over time. Bonferroni pairwise comparisons also demonstrated that the sway velocity in the incongruent condition was significantly greater than that in the congruent condition only during the first time window of platform rotation (R1: *P* = 0.063), with no significant difference between the two subsequent windows (R2: *P* = 0.099; R3: *P* = 0.40).

Finally, we analyzed the sway velocity after the platform was stopped. The results show that the mean sway velocity after platform stop is still significantly larger than the baseline [window *F*(1, 17) = 43.71, *P* = 4.0e-06], indicating that there are some after effects of the disturbance. Further, analysis of the sway velocity for the two windows after platform stopping showed that the sway velocity decreased over time [window *F*(1, 17) = 45.38, *P* = 3.0e-06] and was greater for those experiencing sensory conflict [sensor *F*(1, 17) = 18.99, *P* = 4.3e-04]. In addition, a significant interaction effect was also shown between the two factors [window*Sensor *F*(1, 17) = 9.85, *P* = 0.006]. Bonferroni pairwise comparisons also demonstrated that the sway velocity after the incongruent condition was significantly greater than that in the congruent condition only during the first time window after platform stop (A1: *P* = 0.0019). This also implies that the detrimental effect of sensory conflict on stance stability diminishes over time (A2: *P* = 0.11).

### Changes in brain networks under multisensory stimuli

**FIGURE 4:**
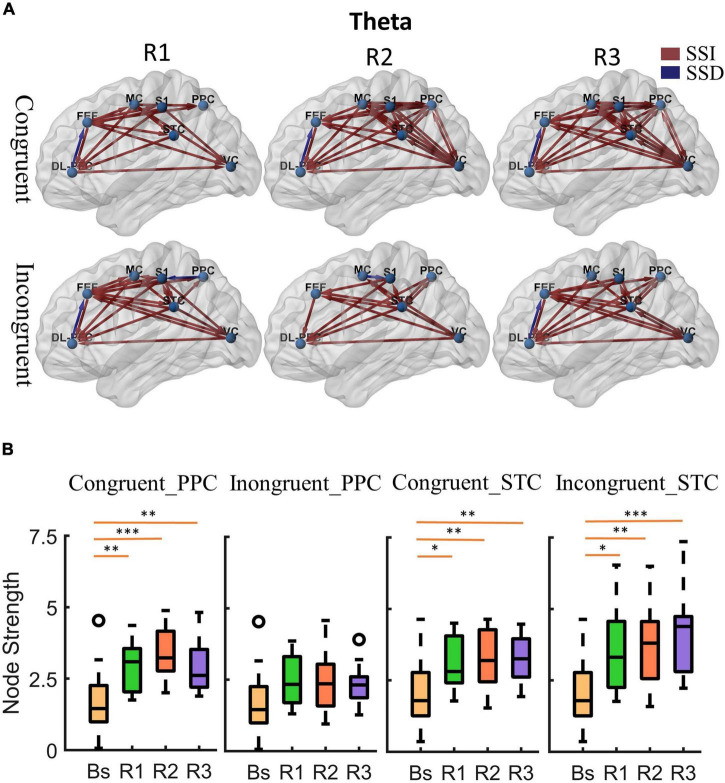
Cortical activity in theta band. **(A)** Changes of theta band cortical connectivity compared to standing baseline in congruent and incongruent conditions. Red lines indicate a statistically significant increase (SSI) in connectivity compared to baseline, and blue lines indicate a statistically significant decrease (SSD) in connectivity compared to baseline (*p* < 0.05). Significances were calculated using paired *t*-tests and corrected by the false discovery rate (FDR) method. The cortical areas of interest are as follows: dorsolateral prefrontal cortex (DL-PFC), frontal eye field cortex (FET), motor cortex (MC), primary somatosensory (S1), posterior parietal cortex (PPC), superior temporal cortex (STC), visual cortex (VC). The arrows represent the direction of information flow. **(B)** Theta band node strength in posterior parietal and superior temporal cortices. Paired *t*-tests were used to determine the difference in node strength between rotating phase (R1, R2, R3) and baseline and were corrected for multiple comparisons using Bonferroni. Congruent _PPC: Node strength of posterior parietal cortex in the congruent condition. Inongruent_PPC: Node strength of posterior parietal cortex in the incongruent condition. Congruent _STC: Node strength of superior temporal cortex in the congruent condition. Inongruent_STC: Node strength of superior temporal cortex in the incongruent condition. The upper and lower error bars of the bins are the upper and lower quartiles of the data, respectively. *p < 0.05, ^**^p < 0.01, ^***^p < 0.001.

**FIGURE 5:**
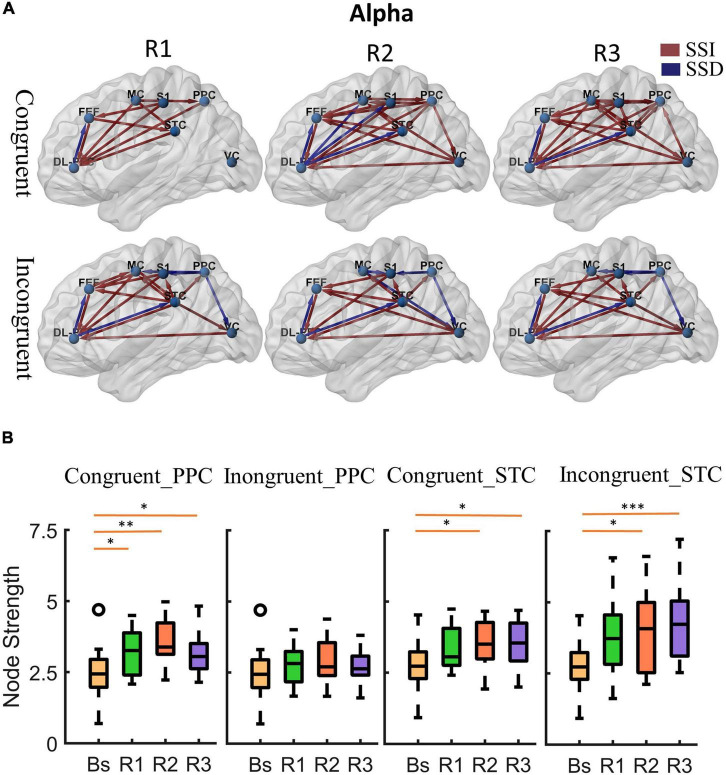
Cortical activity in alpha band. **(A)** Changes of alpha band cortical connectivity compared to standing baseline in congruent and incongruent conditions. **(B)** Alpha band node strength in posterior parietal and superior temporal cortices. *p < 0.05, ^**^p < 0.01, ^***^p < 0.001.

To quantify the status of cortical information flow during equilibrium under multisensory stimuli, we constructed a cortical effective connectivity network using a partial directed coherence function (PDC). Figures 4A, 5A show the significant changes in effective cortical connectivity (p < 0.05, false discovery rate correction) in the rotational stimuli compared to the stationary baseline. The red/blue lines represent effective connectivity during rotational stimulation significantly greater/less than during stationary standing (Supplementary Figures 2, 3 provide the specific *p*-value for each connection pair).

First, at the onset of the rotating stimulus (R1), the effective connections centered in the frontal lobe were significantly increased compared to the baseline. From the first columns of Figures 4A, 5A, we can find that all frontal (DL-PFC, FEF, SMA) regions have enhanced effective connections with other regions in the congruent condition. While in the incongruent condition, in addition to significant activation of the frontal cortical-centered networks compared to the baseline, there was a significant increase in the outflow of information from the superior temporal lobe.

Second, after a period of sustained rotational stimulation (R2, R3), we found a wide activation of cortical networks in the theta band compared to the baseline (as shown in the third and second columns of Figure 4A). In the congruent condition, the effective connections between the visual cortex, motor cortex, somatosensory cortex, posterior parietal lobe, and superior temporal lobe were generally significantly enhanced, forming a network of information interactions centered on parietal-temporal extensions to visual and sensorimotor cortices. In the incongruent condition, we mainly found a significant enhancement of information flow from the temporal lobe to other regions. In addition, the trends were similar in the alpha to theta bands, but the alpha band activated fewer connections during stimulation than the theta band (Figure 5A).

Then, we calculated the node strength in the posterior parietal and superior temporal cortices to further explore the effect of multisensory stimuli. Paired *t*-tests were used to determine the difference in node strength between rotating phase (R1, R2, R3) and baseline and were corrected for multiple comparisons using Bonferroni as shown in Figures 4B, 5B. We could see that the posterior parietal lobe node strength was significantly greater in sensory congruent rotational stimulation compared to the baseline (Theta: *P*_*R1*_ = 0.0013, *P*_*R2*_ = 0.00040, *P*_*R3*_ = 0.0060; alpha: *P*_*R*1_ = 0.034, *P*_*R2*_ = 0.0041, *P*_*R3*_ = 0.019), while there was no significant change during the sensory conflict (Theta: *P*_*R1*_ = 0.091, *P*_*R2*_ = 0.087, *P*_*R3*_ = 0.22; Alpha: *P*_*R1*_ = 0.64, *P*_*R2*_ = 0.14, *P*_*R3*_ = 0.87). In addition, node strength in the superior temporal lobe increased significantly in both of congruent (Theta: *P*_*R1*_ = 0.015, *P*_*R2*_ = 0.0095, *P*_*R3*_ = 0.0016; alpha: *P*_*R*1_ = 0.050, *P*_*R2*_ = 0.035, *P*_*R3*_ = 0.013) and incongruent (Theta: *P*_*R1*_ = 0.010, *P*_*R2*_ = 0.0060, *P*_*R3*_ = 0.00030; alpha: *P*_*R*1_ = 0.057, *P*_*R2*_ = 0.029, *P*_*R3*_ = 0.00090) condition.

**FIGURE 6:**
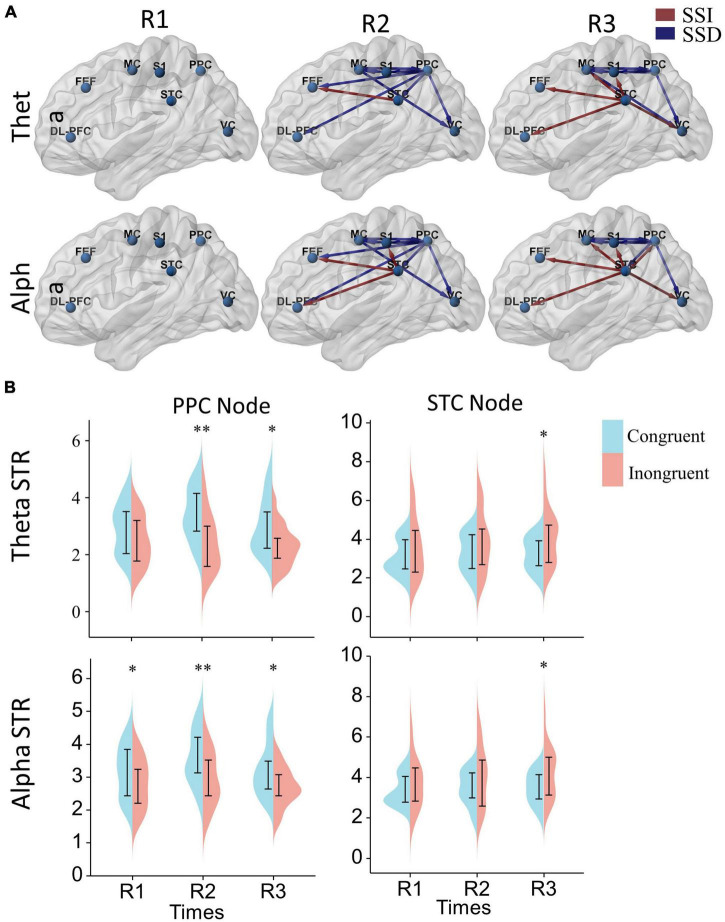
The effect of sensory conflict on cortical effective connectivity networks. **(A)** Significant changes in effective cortical connectivity in the incongruent condition compared to the congruent condition. Red connections indicate significantly stronger connectivity in the conflict condition, and blue connections indicate significantly weaker connectivity in the conflict condition (*p* < 0.05). Significance levels were calculated by paired *t*-test and corrected by the false discovery rate (FDR) method. The arrows represent the direction of information flow. **(B)** Differences in node strength (STR) in the posterior parietal and superior temporal lobes. A two-way repeated measures ANOVA with Bonferroni *post-hoc* tests to assess the effects of the time window and sensory condition on node strengths. *p < 0.05, ^**^p < 0.01.

Finally, we analyze the difference in the effective cortical network between the congruent and incongruent conditions (p < 0.05, false discovery rate correction), as shown in Figure 6A, where the red/blue lines represent the effective connections in the incongruent condition significantly greater/less than the congruent condition (Supplementary Figure 4 provides the specific *p*-value for each connection pair). First, At the beginning of the rotational stimulus (R1), there are no effective connections with significant differences between the congruent and incongruent conditions. Then, after a while (R2, R3), a similar phenomenon was observed for both alpha and theta bands; that is, the network of effective connections centered in the parietal lobe is significantly weaker in the incongruent condition compared to the congruent condition, but the information flow from the superior temporal lobe is significantly stronger.

Further, to analyze the differences in node strengths between the congruent and incongruent conditions. We used two-way repeated measures ANOVA with Bonferroni *post-hoc* tests to assess the effects of the time window and sensory condition on node strengths, as shown in Figure 6B. We can also find that the node strengths of the posterior parietal lobe in the congruent condition are significantly greater than those in the incongruent condition [Theta: sensor *F*(1, 17) = 15.25, *P* = 0.0011, *P*_R2_ = 0.0045, *P*_R3_ = 0.021; alpha: sensor *F*(1, 17) = 8.59, *P* = 0.0093, *P*_R1_ = 0.032, *P*_R2_ = 0.0045, *P*_R3_ = 0.019], while the node strengths of the temporal lobe in the incongruent condition are significantly greater than those in the congruent condition [Theta: sensor *F*(1, 17) = 4.79, *P* = 0.043, *P*_R3_ = 0.015; alpha: sensor *F*(1, 17) = 4.56, *P* = 0.048, *P*_R3_ = 0.017]. In terms of time window factor, there was no statistically significant difference in the node strengths of both posterior parietal [Theta: window *F*(2, 34) = 1.22, *P* = 0.31; alpha: window *F*(2, 34) = 2.51, *P* = 0.096] and superior temporal lobes [window *F*(2, 34) = 0.80, *P* = 0.46; alpha: window *F*(2, 34) = 0.87, *P* = 0.43].

## Discussion

In the present study, we combined a rotating platform and a VR headset to control the participants’ physical and visual motion states. Then we analyzed the effective connectivity dynamics changes and postural stability of subjects under visual and actual motion congruent or incongruent. We mainly find that: (1) Sensory conflict had a significant detrimental effect on postural stability. However, human can adapt to this detrimental effect over time. (2) The recovery of humans standing balance under sensory conflict was associated with changes in the cortical network. At the onset of the rotational stimulus, sensory congruent and incongruent rotational stimuli had similar effects on brain networks. And after a while, synchronization with the restoration of balance control, congruent and incongruent stimuli had broad and different effects on cortical networks.

### The role of frontal cortex

In the present study, we found that although sensory conflict significantly negatively affected participants’ balance control at the beginning, this effect diminished and disappeared over time. This suggests that the humans has an adaptive capacity to balance challenges in sensory conflict. Furthermore, we found that effective connectivity networks centered in the frontal cortex were significantly activated during the beginning of balance challenges and persisted throughout the task, regardless of the sensory condition.

Actually, the frontal cortex has long been widely known to play an important role in reasoning, decision-making, and adaptive behavior (Mihara et al., 2008). Also, the frontal lobes are thought to have an important role in balance control; several studies have shown increased activation of the frontal lobes during balance perturbations (Clark et al., 2014; Fujita et al., 2016). And the intensity of frontal activation is positively correlated with task difficulty and inversely correlated with balance performance (Lu et al., 2015; Osofundiya et al., 2016). In addition, in multisensory processing, several studies suggest that the frontal lobes may play a role in multisensory causal inference, participating in the arbitration of integration and dissociation, and may be critical to the brain’s flexibility in multisensory information processing (Rohe and Noppeney, 2015; Cao et al., 2019). Here, We also found that rotational stimulation first activated the frontal cortex. Then synchronized with the restoration of balance control, in congruent and incongruent conditions, the sensory systems of the brain connectivity network showed integrated and dissociated connectivity, respectively. This may be a crucial mechanism for humans stance balance control during complex and variable environments.

### Multisensory integration network

During balance control under congruent conditions, we found a general increase in information interaction between the posterior parietal, superior temporal, motor, somatosensory, and visual cortices in the theta and alpha band. It forms a network of information interactions centered on parietal-temporal extensions to visual and sensorimotor cortices, which may be a vital neural process for multisensory integration in the stand balance control.

Actually, many studies have highlighted the important role of the posterior parietal and superior temporal lobes in sensory integration and suggest that multisensory information may be combined in it (Calvert, 2001; Beauchamp et al., 2004; Macaluso and Driver, 2005). By using neuroimaging, multiple cortical regions in the human brain’s parietal and temporal lobes can respond to stimuli of more than one modality (Bremmer et al., 2001; Macaluso and Driver, 2001), which agrees with single-cell recordings of cortical heteromodal neurons in these regions in other primates (Graziano et al., 2002). Further, neurophysiological studies in primates have shown that the activity of some multisensory neurons in the superior temporal and posterior parietal lobes is regulated by changes in the reliability of cues on trial, similar to the dynamic adjustment of psychophysical weights, which are a hallmark of sensory weighting integration (Schaafsma and Duysens, 1996; Bremmer et al., 2002; Gu et al., 2008; Morgan et al., 2008; Fetsch et al., 2011). In addition, oscillations and synchronization in the alpha spectrum and below are increasingly understood as a component of neuronal communication and integration. Theta oscillations are also suggested to coordinate different brain functions (e.g., updating motor planning after somatosensory input-the sensory-motor integration hypothesis) (Bland and Oddie, 2001). Jensen and colleagues describe theta oscillations can be used as a carrier wave for information transfer between brain regions (Jensen and Colgin, 2007). Further findings from performing a pathfinding task in a VR environment suggest that the mechanism of sensorimotor integration is guided by theta oscillations (Caplan et al., 2003).

Taken together, during balance control under congruent physical-visual stimuli, it is not surprising that we found network activation centered on the parietal-temporal lobe in the theta and alpha band, which may reflect the cortical information interaction process of multisensory integration.

### Flexible multisensory processing under sensory conflict

The effective connectivity of cortical networks under sensory conflict significantly differed from control. First, during the conflicting sensory condition, we found a general paucity of information flow across cortical areas involved in the sensory integration network centered in the parietal-temporal lobe. This may reflect a flexible multisensory interaction strategy, and separate processing of sensory cues may be a general response to sensory information mismatch. In addition, during sensory cue conflict, we also found a significant increase in the influence of the superior temporal lobe on other cortical regions, and the posterior parietal centered cortical network was significantly diminished. This may reflect functional specialization of the superior temporal and posterior parietal lobes.

Although no previous studies have proposed specific functional differences between the superior temporal lobe and the posterior parietal lobe, it has been shown as early as in primate neurophysiological studies that the posterior parietal and superior temporal lobes differ in their response properties to sensory stimuli (Chen et al., 2013; Cao et al., 2019). It has been found that there are roughly equal populations of neurons in the superior temporal lobe that are sensitive to congruent or incongruent stimuli, whereas more populations of neurons in the posterior parietal lobe are sensitive to congruent stimuli (Yang et al., 2011; Chen et al., 2013). It is well known that congruent neurons may be the neural basis of sensory integration, but the role of opposite neurons has puzzled neuroscience for a long time (Morgan et al., 2008; Fetsch et al., 2011). In recent years, some researchers have suggested that combining opposite and congruent neurons may help people make multisensory causal inferences for the flexible processing of multisensory information. Signals were attributed to the same event driving sensory integration when congruent cells are more active. In contrast, when opposite cells are more active, they are attributed to different events for independent multisensory processing (Zhang et al., 2019; Badde et al., 2021; Rideaux et al., 2021).

These studies’ conclusions are mutually supportive of our study’s results. The posterior parietal lobe is the center of sensory integration, and its information interaction with other cortical is enhanced mainly when sensory cues are congruent. In contrast, the superior temporal lobe also plays a role in the arbitration of sensory integration and segregation, and it should be activated in both incongruent and congruent stimuli but may play a greater role in the sensory conflict.

## Conclusion

The present study analyzed the effective connectivity dynamics changes during standing balance control under visual-actual motion congruent and incongruent. We found that the recovery of humans standing balance was associated with changes in the cortical network and synchronized with the recovery of the balance control congruent and incongruent stimuli had broad and different effects on cortical networks. During sensory congruent, information interactions among sensory systems are significantly enhanced and integrated. While during the sensory conflict, information pathways between visual and sensorimotor systems are almost disconnected, and only the influence of the superior temporal lobe on other cortical regions significantly increases. These results may reflect a flexible multisensory interaction strategy critical for human posture balance control in complex and changing environments.

## Data availability statement

The raw data supporting the conclusions of this article will be made available by the authors, without undue reservation.

## Ethics statement

The studies involving human participants were reviewed and approved by the Ethics Committee of Zhejiang University Psychological Science Research Center permitted our experiment (issued no. 2020-003). The patients/participants provided their written informed consent to participate in this study. Written informed consent was obtained from the individual(s) for the publication of any potentially identifiable images or data included in this article.

## Author contributions

GW and YY were involved in study design, article writing and revision, and data collection. ZH was involved in study design and article revision. XL was involved in experimental data collection and study design. JW and JL were involved in study design, article revision, organization, and equipment provision. All authors agreed to be accountable for the content of the work.
