# Supplementary material for: Dynamic changes of brain networks during standing balance control under visual conflict
Source: Front Neurosci. 2022 Oct 5;16:1003996. doi: 10.3389/fnins.2022.1003996 (PMC9581155; doi:10.3389/fnins.2022.1003996)
Supplement: Supplementary file 1 [file Data_Sheet_1.docx]

In the present study, in addition to the rotational stimuli we designed, there was also a weak vibration stimulus exerted on the participants because of the performance deficits of the rotating platform. As shown in Supplementary Figure 1A, we measured the cop data of a 50kg weight (middle of the figure) during the experimental procedure and compared it with the participants (left of the figure). From the cop trajectory of the weights, we know that the rotating platform has been vibrating weakly after starting, and there will be a more violent vibration once in every half rotation cycle. And this periodic vibration carried the periodic postural adjustment of the human body, with the result that we saw that the cop trajectory of the healthy participants also showed an oscillation pattern consistent with the rotation cycle.

Further, we perform mean sliding filtering on the cop of the weight, and we may see that it has an oscillation pattern consistent with the human body (the right side of the figure). Previous studies have found a similar phenomenon(1). A saturation point is typically observed, beyond which an increase in the intensity of the sensory stimulus causes no further increases in postural sway amplitude (e.g(2, 3)). The gestural control system can re-weight its available sensory inputs by reducing the weight of unreliable or high intensity sensory inputs in order to optimize gestural control in changing sensory environments(4, 5).

Of course, because this interference is equal for everyone, we do not think it affects the scientific nature of our results. In fact, it is equivalent to adding an additional vibrational interference to our experiment, which increases the difficulty of maintaining the standing balance. However, the experiment with the weights reveals that this vibration is consistent in each cycle, and the speed of the weights' wobble does not decay over time (Figure1B). This suggests that the variation of the human sway speed is indeed a mechanism of postural control. In addition, outside of the programmed scene movement, normal head movements are also picked up by the IMU in the VR headset and change the perspective of the virtual scene accordingly. And we set up the scene of sensory conflict that only changed the rotational movement's direction. Therefore, the vibration of the rotation platform does not change our sensory mode but only adds a congruent sensory input outside the conditions we set up.

Reference

1. Oie KS, Kiemel T, Jeka JJ. Multisensory Fusion: Simultaneous Re-Weighting of Vision and Touch for the Control of Human Posture. *Cognitive Brain Res* (2002) 14(1):164-76. doi: Pii S0926-6410(02)00071-X

Doi 10.1016/S0926-6410(02)00071-X.

2. Peterka RJ, Benolken MS. Role of Somatosensory and Vestibular Cues in Attenuating Visually Induced Human Postural Sway. *Exp Brain Res* (1995) 105(1):101-10.

3. Van Asten W, Gielen C, Van Der Gon J. Postural Adjustments Induced by Simulated Motion of Differently Structured Environments. *Exp Brain Res* (1988) 73(2):371-83.

4. Fetsch CR, Turner AH, DeAngelis GC, Angelaki DE. Dynamic Reweighting of Visual and Vestibular Cues During Self-Motion Perception. *J Neurosci* (2009) 29(49):15601-12. Epub 2009/12/17. doi: 10.1523/JNEUROSCI.2574-09.2009.

5. Mahboobin A, Loughlin P, Atkeson C, Redfern M. A Mechanism for Sensory Re-Weighting in Postural Control. *Med Biol Eng Comput* (2009) 47(9):921-9. Epub 2009/03/28. doi: 10.1007/s11517-009-0477-5.


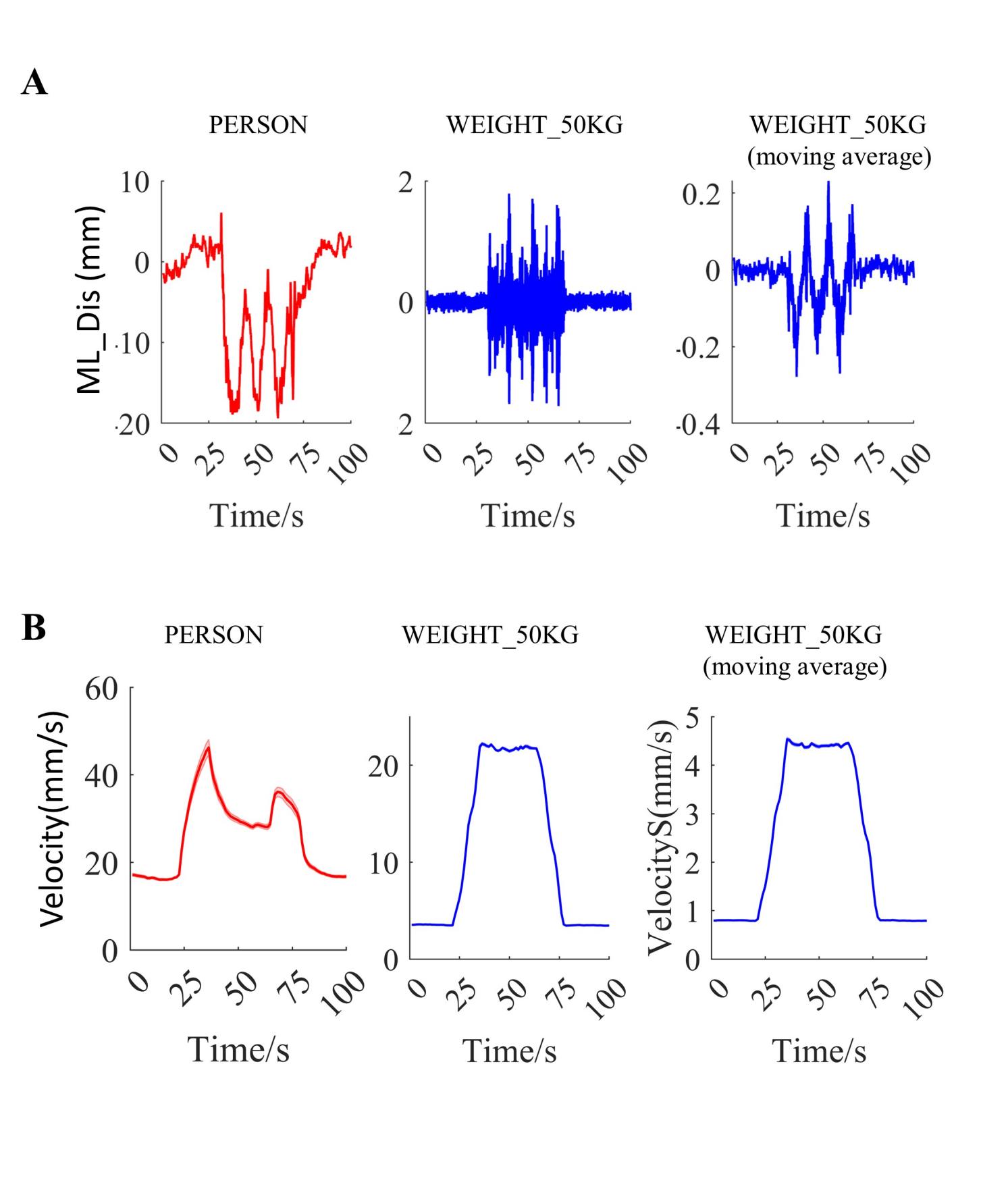


**Supplementary Figure 1.** A: Mean displacement trajectory of AP center of pressure (COP). On the left of the graph is the average shaking trajectory of 18 healthy participants. In the middle of the picture, a 50kg weight was used instead of young people to conduct 9 experiments. On the right side of the figure, a sliding mean filter is performed on the swaying trajectory of the weight, the window is 30 sampling points, and the step size is one sampling point. B: Trend of mean COP sway velocity over time.


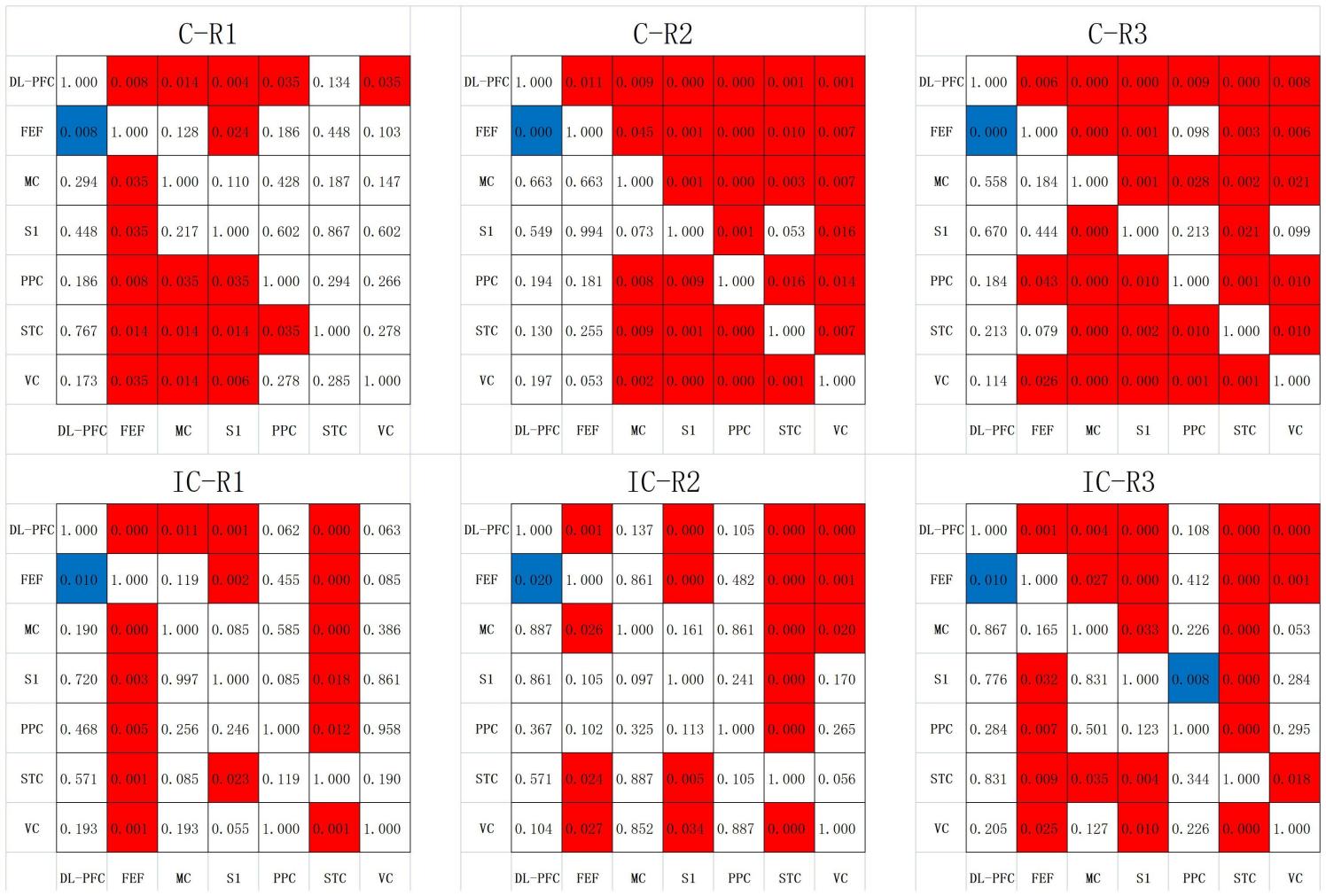
Supplementary Figure 2. Significantly different connectivity from the baseline in the theta band brain network. Significances were calculated using paired t-tests and corrected by the false discovery rate (FDR) method. The values in the image represent the p-values obtained from statistical tests. Red cells represent a significant increase for that connection pair and blue represents a significant decrease (P<0.05). IC: incongruent, C: congruent.


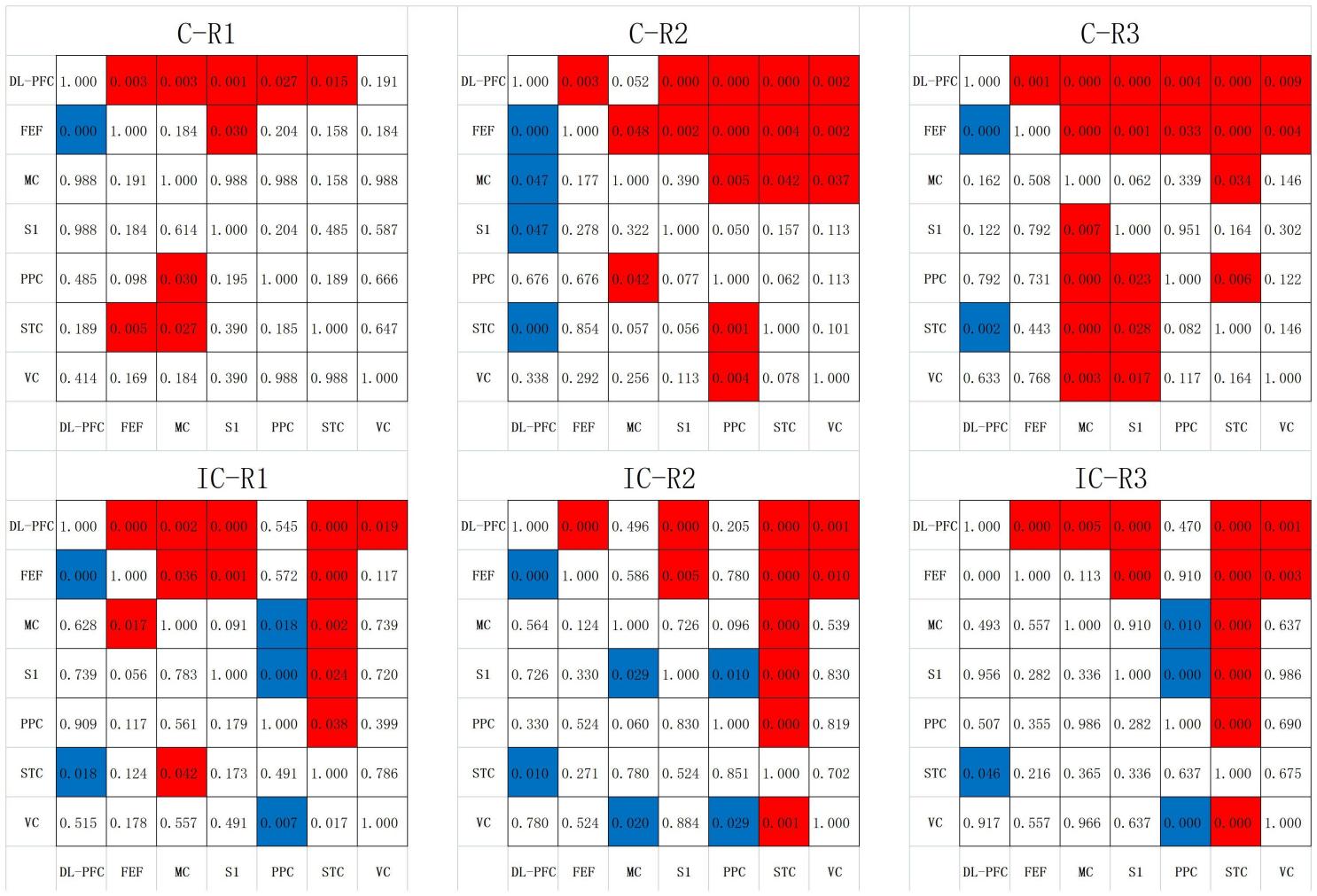


**Supplementary Figure 3.** Significantly different connectivity from the baseline in the alpha band brain network. IC: incongruent, C: congruent.


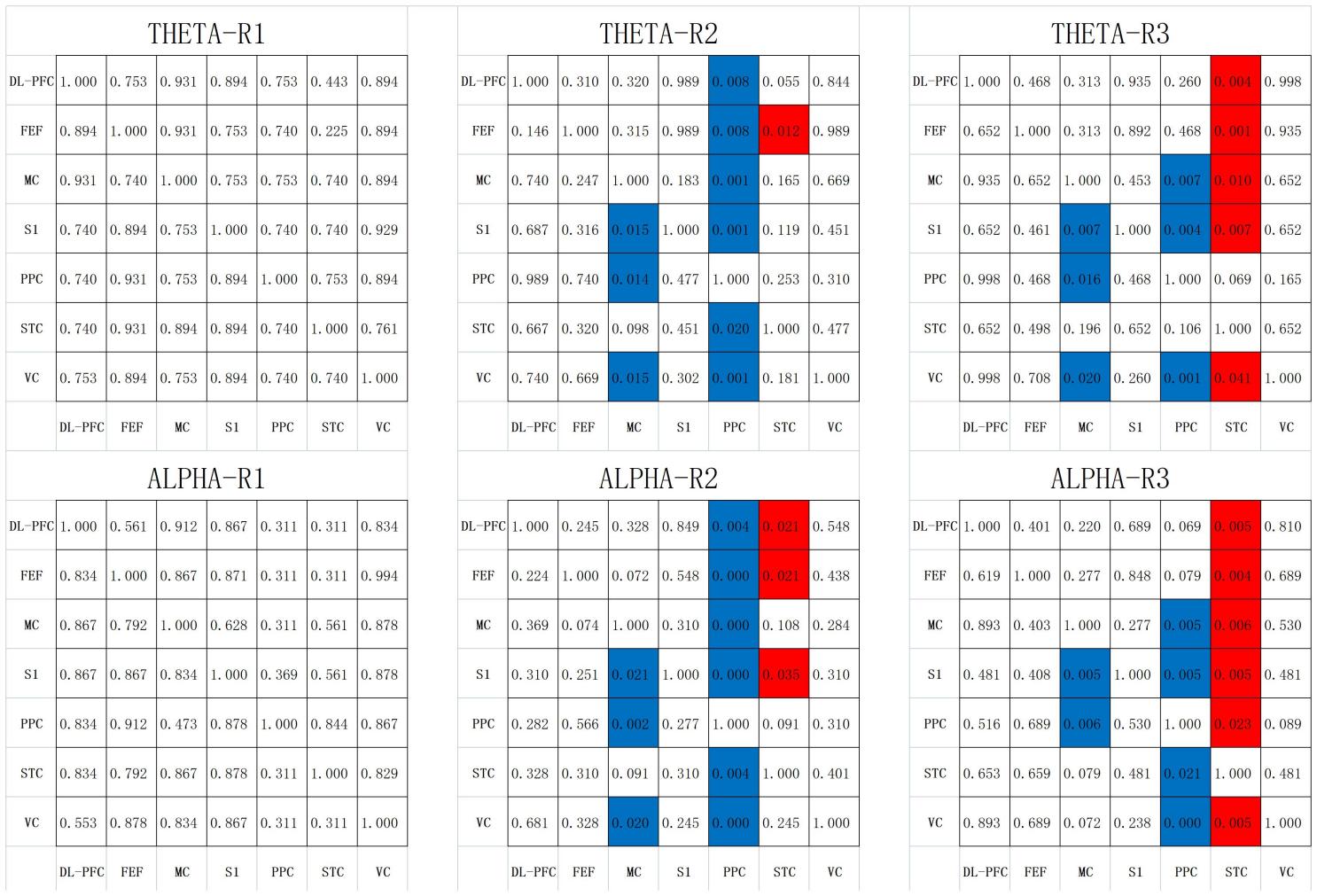


**Supplementary Figure 4** Significant changes in effective cortical connectivity in the incongruent condition compared to the congruent condition. Significances were calculated using paired t-tests and corrected by the false discovery rate (FDR) method. The values in the image represent the p-values obtained from statistical tests. Red cells indicate significantly stronger connectivity in the conflict condition, and blue cells indicate significantly weaker connectivity in the conflict condition (p<0.05). IC: incongruent, C: congruent.
